# Urban PM_0.2_ Affects Astrocytes by Inducing Xenobiotic and Oxidative Stress Responses

**DOI:** 10.1007/s12035-026-06232-w

**Published:** 2026-10-02

**Authors:** Liudmila Saveleva, Alexey Afonin, Mariia Ivanova, Michal Sima, Zdenek Krejcik, Tarja Malm, Pasi I. Jalava, Pavel Rössner, Katja M. Kanninen

**Affiliations:** 1 A.I. Virtanen Institute for Molecular Sciences, University of Eastern Finland, Kuopio Campus, Neulaniementie 2, P.O. Box 1627, 70211 Kuopio, Bioteknia Finland; 2 Department of Toxicology and Molecular Epidemiology, Institute of Experimental Medicine CAS, Prague, Czech Republic; 3 Department of Environmental and Biological Sciences, University of Eastern Finland, 70211 Kuopio, Finland

**Keywords:** PM, Air pollution, Astrocytes, Glia, Proteomics, Transcriptomics

## Abstract

Air pollution is the largest environmental risk factor contributing substantially to global mortality rates. Several studies demonstrated that pollutant exposure leads to neuroinflammation and oxidative stress, yet little is known about the effects induced in specific brain cell types. Here, we aimed to decipher how nanoscale particulate matter (PM_0.2,_ an aerodynamic diameter < 0.2 µm) collected from urban air affects a central element of the brain's homeostatic system – the astrocytes, via 24-h exposure of primary astrocyte cultures to PM_0.2_ at a final concentration of 50 µg/ml. We combined transcriptomic and proteomic profiling to elucidate astrocyte-intrinsic responses to PM. PM_0.2_ exposure altered the expression of 2217 mRNA, with the robust induction of xenobiotic metabolism, the Nrf2 oxidative stress response, and glutathione-mediated detoxification. Strong upregulation of cytochrome P450 genes, glutathione S-transferase superfamily, *Nqo1, Ahrr* and *Slc17a8* was detected. Exploratory proteomic analysis identified 354 proteins with nominal evidence of altered abundance, 142 of which showed corresponding significant mRNA changes. The cross-platform-supported candidates were consistent with coordinated activation of xenobiotic-metabolism and redox-homeostasis pathways. Reanalysis of published RNA-seq datasets showed that, in contrast to PM-exposed microglia, which predominantly activate pro-inflammatory signaling pathways, astrocytes preferentially engage detoxification and antioxidant programs, while sharing a subset of stress and signaling pathways with the PM-exposed cerebral cortex in in vivo model. Our results provide insight into astrocyte-specific responses to urban air pollutant exposure and allow a better understanding of the processes activated by PM exposure in the brain.

**Supplementary Information:**

The online version contains supplementary material available at https://doi.org/10.1007/s12035-026-06232-w.

## Introduction

The latest World Health Organization reports highlight that most of the global population breathes polluted air. The impact of poor ambient air quality on global health is detrimental [1], however, is constantly overlooked. Air pollution exposure results in millions of premature deaths every year. The complex mixture of gases, particulate matter (PM), and other contaminants that make up air pollution has negative effects on human health, including the brain [2]. The health effects of ambient PM based on epidemiological and experimental evidence were summarized in a review by Li et al. [3]. Acquired data pinpoint the negative effects of PM exposure on brain health and correlate poor air quality with neurodegenerative disease risk and cognitive decline. Even though all types of PM were reported to affect human health, the detrimental effects of the nanoscale fraction of PM on the brain have recently accumulated increasing evidence [4–7]. In vitro studies reported a size-dependent cytotoxic impact of PM [5]. For PM_0.2_, the diameter of less than 0.2 µm results in a big surface area per mass that contributes to increased toxicity of this fraction when compared to larger PM.

In addition, a small diameter allows for easier translocation of the particles to the brain. Ultrafine PM fraction (diameter < 0.1 μm) was found in the exposed brain in both experimental models [4, 8–11], and humans [12, 13]. Furthermore, Qi et al. characterized fine particles (diameter of 0.1–2.5 μm) in the human brain and proved their translocation through both direct route of entry, like the olfactory nerve route, and indirect, systemic translocation [14]. The exact molecular mechanisms activated by PM exposure in the brain and the response of different cells composing the brain to the exposure remain to be investigated.

Activation of glial cells, along with an antioxidative stress response, remains one of the most reported consequences of PM exposure in experimental studies. Although most work has focused on microglia, recent evidence indicates that PM also induces reactive astrocyte phenotypes in experimental models [15]. Astrocytes are the most abundant neuroglial cells [16] and are essential for maintaining brain homeostasis and regulating neuronal activity [17]. Astrocytes have a wide repertoire of pivotal functions in the brain, such as maintaining blood–brain barrier (BBB) integrity and neurotransmitter release, supplying nutrients and precursors to neurons, ion buffering, and modulating the immune response in the brain [18]. Given that all these processes may be affected by PM exposure, which can result in changes in astrocyte states and overall brain homeostasis, it is important to better understand the processes behind PM-related effects in astrocytes. The reactive state of astrocytes in PM-exposed humans [12, 19] and animal models [4, 8, 20, 21] was reported previously. An increase in the marker of the reactive state of astrocytes – glial fibrillary acidic protein – is reported in numerous in vitro and in vivo data; however, the data on exact molecular and cellular effects remain limited [22].

## Materials and Methods

### Primary Astrocyte Culture

We prepared primary adult astrocyte cultures as described previously by us in [23] and in methodological studies in [24] and [25]. In brief, cortices were isolated from ten 4–6-month-old female C57BL6J mice after transcardial perfusion with heparinized saline. The cortices were homogenized with an enzyme-based procedure and the Adult Brain Dissociation Kit (Miltenyi Biotech, Germany) according to the manufacturer's instructions. Cells were cultured in a DMEM/F-12 medium containing 10% heat-inactivated fetal bovine serum, 1% penicillin–streptomycin, and GlutaMAX™ supplement (all from Gibco, USA). Primary cells from each mouse were cultured in separate plates. RNA-seq and DIA-proteomic profiling were performed using separate, independent cohorts of primary astrocyte cultures derived from different individual mice (n = 5 biological replicates per treatment group for each platform). Biological replicates derived from unique mice were divided into technical replicates by culturing cells in different wells. On day 10 in vitro (DIV) of culturing cells as primary mixed glia cultures, microglia were depleted from the culture with magnetic sorting with CD11b MicroBeads (Miltenyi Biotech, Germany), and only the CD11b-negative population of astrocytes was kept in the culture. The procedure of isolation and culture of pure adult mouse astrocytes was previously described in [24] and [25]. On DIV 21, the cell culture media was replaced with media containing either PM_0.2_ suspension or DMSO and water (vehicle control). Wells on the plate originated from each mouse were divided into the vehicle and PM_0.2_ group, with 5 mice (individual biological replicates) per treatment group for each assay. After 24 h, cells were collected for subsequent analysis.

Although primary astrocyte cultures purified by microglial depletion using CD11b-coated beads are a well-characterized research model [24, 25], we performed an additional computational validation by comparing our dataset with a previously published transcriptomic profiling of mouse microglia and astrocytes [26]. PCA revealed that expression signatures of our samples were more similar to reference astrocyte transcriptomes than to microglial transcriptomes (Supplementary Figure S2), reconfirming sample identity.

### PM_0.2_ Exposure

The PM particles used in the study were previously characterized in [27] and [28]. Only PM with a diameter of less than 200 nm were used in this study. We used PM_0.2_ that was collected during the nighttime from the Nanjing Xianlin campus, China, in the pre-intervention campaign in May–June 2014. On the exposure day, PM_0.2_ samples were suspended in 10% v/v sterile DMSO (Sigma-Aldrich, USA) in sterile water (Baxter, USA) as a 100 × stock (5 mg/ml). A mixture of only sterile water and sterile DMSO was used as a vehicle control. Then, we used an ultrasonic water bath to sonicate PM_0.2_ samples and vehicle controls for 30 min. Freshly sonicated pollutants or vehicle were administered to cells in a culture medium at final concentrations of 50 µg/ml. PM_0.2_ concentration was selected from preliminary MTT dose–response testing as the dose causing a slight decrease in cell viability without significant cell toxicity (Sup. Fig. S1; viability > 80% of its vehicle at working dose).

### mRNA Sequencing Analysis

#### RNA Isolation

On the last day of the experiment, cells were washed once in DPBS (Gibco, USA) for removal of media and then collected directly in TRI Reagent (Sigma-Aldrich, USA), followed by total RNA isolation according to the manufacturer’s instructions. NanoDrop 2000 (Thermo Fisher Scientific, USA) was used for determining the concentration and purity of all RNA samples. RNA samples with 260/280 ratios higher than 1.8 and RNA integrity values higher than 8.0 were used for analyses.

#### Library Preparation and RNA Sequencing NGS

Qubit 4 fluorometer and the HS RNA kit (both from ThermoFisher Scientific, USA) were used to determine extracted RNA amounts. mRNA was isolated with NEBNext Poly(A) mRNA Magnetic Isolation Module (New England Biolabs, USA). mRNA libraries were prepared with NEBNext Ultra II Directional RNA Library Prep kit and NEBNext Multiplex Oligos for Illumina (both New England Biolabs, USA) in accordance with the producer's guidelines; 200 ng of total RNA was used. A 1xdsDNA HS kit (Thermo Fisher Scientific, USA) was used to measure the concentration of mRNA libraries with the Qubit 4 fluorometer. mRNA libraries were then analyzed with the Fragment Analyzer and the HS NGS Fragment Kit (both Agilent Technologies, USA). Pair-end (2 × 60 cycles) sequencing was performed with the NovaSeq 6000 instrument and NovaSeq 6000 S1 Reagent Kit v1.5 (all Illumina, USA). The quality of the reads was checked using FastQC and Multiqc [29].

In addition to our data, we reanalysed publicly available RNA-seq datasets from previously published studies [30] and [31] downloaded from the NCBI GEO archive under accession numbers GSE183098 and GSE142066. Additional datasets used in this paper are listed in Supplementary File S1 (Sup. File S1). All the downloaded RNA-seq samples were reanalysed using the pipeline used for the original data for this paper.

For the analysis, all the reads were trimmed, and the adapter sequences were removed. The mouse genome mm39 was used to map the mRNA-seq reads. The trimmed sequences were mapped onto the genome using STAR (v. 2.7.10) [32]. The resulting bam files were analysed using the featurecounts script from the subread package (v 2.0.2) [33]. Principal component analysis (PCA) was used for sample clustering; the results are presented in Sup. Fig. S2, S3. The DGE analysis was performed using the DeSeq2 package (v. 1.38.3) [34] the p-values were adjusted using the BH method, reported as q-values. All the data analysis, statistics and figure preparation were performed in the R programming environment (4.2.1).

### Analysis of Protein Expression

#### Protein Precipitation and Digestion

Samples were reduced with 10 mM D, L-dithiothreitol, and alkylated with 40 mM iodoacetamide, followed by overnight digestion with sequencing-grade modified trypsin (Promega). After digestion, peptides were desalted with a Sep-Pak tC18 96-well plate (Waters) and evaporated to dryness. Samples were dissolved in 0.1% formic acid, and peptide concentration was determined with a NanoDrop device. For DIA analysis, 800 ng of peptides were injected and analysed in random order. Wash runs were submitted between each sample to reduce the potential carryover of peptides.

##### LC–ESI–MS/MS

The LC–ESI–MS/MS analysis was performed on a nanoflow HPLC system (Easy-nLC1200, Thermo Fisher Scientific) coupled to the Orbitrap Exploris 480 mass spectrometer (Thermo Fisher Scientific, Bremen, Germany) equipped with a nano-electrospray ionization source. Peptides were first loaded on a trapping column and subsequently separated in-line on a 15 cm C18 column (75 μm × 15 cm, ReproSilPur 3 μm 120 Å C18-AQ, Dr Maisch HPLC GmbH, Ammerbuch-Entringen, Germany). The mobile phase consisted of water with 0.1% formic acid (solvent A) or acetonitrile/water (80:20 (v/v)) with 0.1% formic acid (solvent B). A 120 min gradient was used to elute peptides (60 min from 5 to 21% solvent B, followed by 48 min from 21 to 36% solvent B, and 6 min from 36 to 100% solvent B, followed by a 6 min wash stage with solvent B). Samples were analysed by a data-independent acquisition (DIA) LC–MS/MS method. MS data was acquired automatically by using Thermo Xcalibur 4.6 software (Thermo Fisher Scientific). In a DIA method, a duty cycle contains one full scan (400–1000 m/z) m/z) and 40 DIA MS/MS scans covering the mass range 400–1000 with variable-width isolation windows.

#### Protein Identification and Quantification Analysis

Data analysis consisted of protein identifications and label-free quantifications of protein abundances. Data were analysed by Spectronaut software (Biognosys; version 20.4.2). DirectDIA approach was used to identify proteins, and label-free quantifications were performed with MaxLFQ. Main data analysis parameters in Spectronaut were: (i) Enzyme: Trypsin/P; (ii) up to 2 missed cleavages (iii) Fixed modifications: Carbamidomethyl (cysteine); (iv) Variable modifications: Acetyl (protein N-terminus) and oxidation (methionine); (v) Precursor FDR Cutoff: 0.01 (vi) Protein FDR Cutoff: 0.01 (vii) Quantification MS level: MS2 (viii) Quantification type: Area under the curve within integration boundaries for each targeted ion (iv) Protein database: Swiss-Prot 2023_01 *Mus Musculus* [35], Universal Protein Contaminant database [36]; and (v) Normalization: global median normalization (based on RT-dependent local regression model described by [37]. All the peptides were used for quantification.

#### Proteome Data Analysis

Data pre-processing and statistical analyses were performed using R (version 4.2.1). PCA was performed to assess the general quality of the dataset. Sample normalisation was performed using the medianCentering method from the proBatch package [38]. The proteins identified as contaminants and proteins with values present in at least 3 samples in either group were kept for further analysis. Missing values remaining in the dataset were imputed using the Sample Minimum method [39]. For the differential abundance, the DeqMS package was used [40]. The p-values were adjusted using the Benjamini–Hochberg method; however, due to the low number of samples in each group, nominal p-values were also investigated, as suggested by [41]. Because no proteins met the predefined false-discovery-rate threshold, proteins with nominal p < 0.05 were retained for exploratory and integrative analyses and are referred to throughout as nominally differentially abundant proteins. Protein-level findings were prioritized when the corresponding gene was significantly differentially expressed in the independent RNA-seq dataset and showed a concordant direction of change.

### Pathway Analyses

QIAGEN Ingenuity Pathway Analysis Software (QIAGEN IPA, QIAGEN Inc., USA) [42] was used for canonical pathway analysis and generating figures for DEGs in all datasets analysed and for differentially abundant proteins (DAPs); no log-fold change filters were applied to the features used for the enrichment. Pathway enrichment and protein-network analyses based on the nominally DAP set were considered exploratory and were interpreted solely in conjunction with the RNA-seq data. The z-scores were reported by the IPA software. StringDB (v 12.0) was used to construct the protein interaction network for the proteomics data [43]. The MCL algorithm was used to create protein clusters [43]. Cross-platform integration was performed across independently generated transcriptomic and proteomic datasets. The STRING network visualization was performed using Cytoscape (v. 3.10.3) and OmicsVisualiser (v. 1.3.1). For the integrated proteomic pathway analysis shown in Fig. 3C, IPA was performed specifically on the 142 cross-platform-supported mRNA-protein pairs.

## Results

### Exposure to PM_0.2_ Affects the Astrocyt*e *Transcriptome

**Fig. 1:**
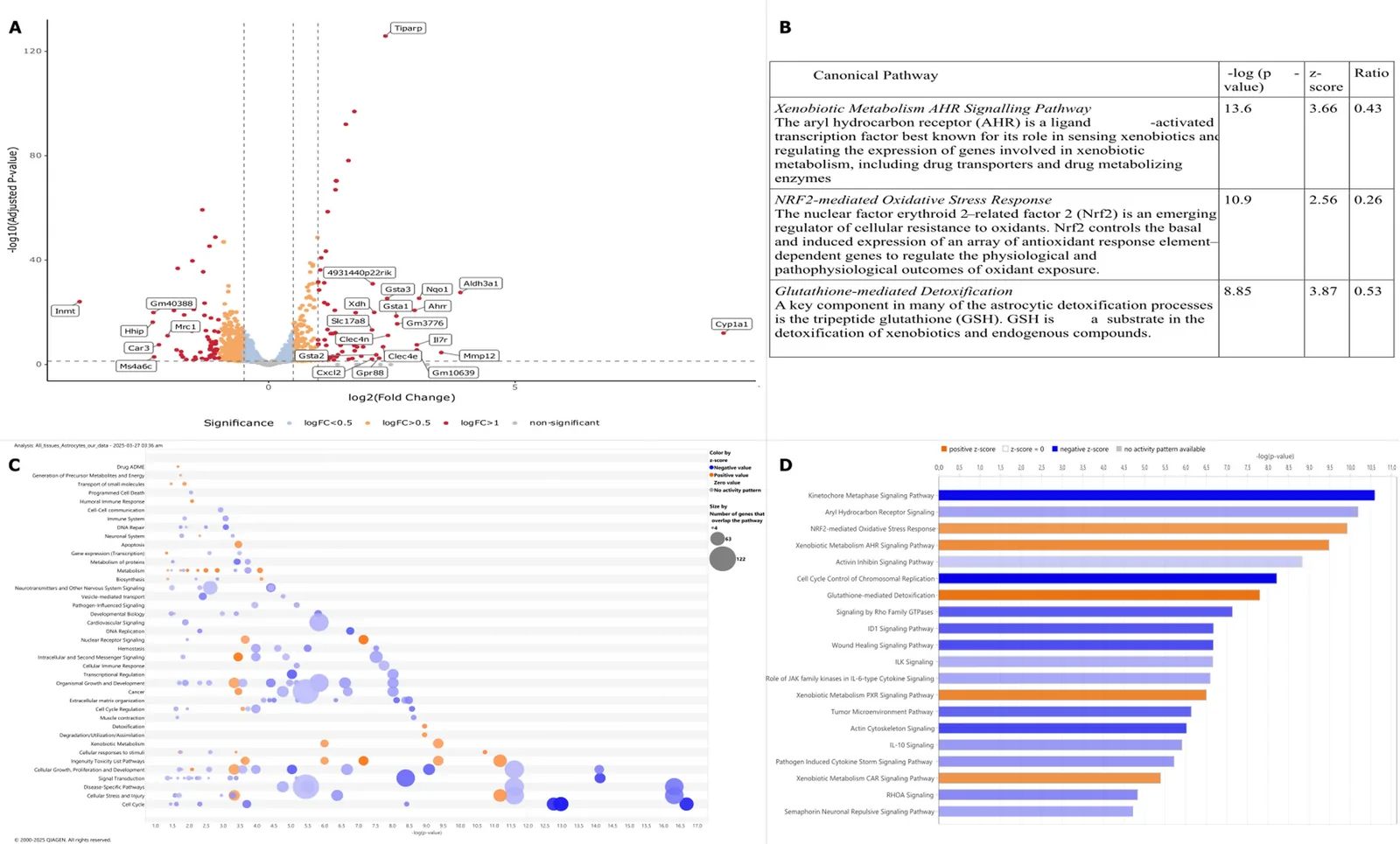
**A** Volcano plot depicting differentially expressed genes (DEGs) following exposure. Insignificant DEGs are shown as grey dots, while significant DEGs are color-coded based on log2 fold change (logFC): blue for |logFC|< 0.5, orange for 0.5 ≤|logFC|< 1 and red for |logFC|≥ 1. The labelled genes represent selected DEGs of interest.** B** Table with the top three deregulated pathways in PM_0.2_-exposed astrocytes with q-value, z-score and ratio score. **C** Bubble chart depicting the top pathway categories (Y-axis) altered in astrocytes after PM_0.2_ exposure, with Z-score value for each functional category (X-axis). Only pathways with an absolute Z-score of more than 1 are presented. The plot is sorted by the -log(q-value); the bubble size indicates the ratio of overlapping genes to the total number of genes in the pathway, while bubble colour depicts the gradient of pathway activation, with downregulated pathways depicted in blue and upregulated pathways depicted in orange. **D** Top 20 canonical pathways deregulated by PM_0.2_ exposure in astrocytes provided by IPA software, sorted by -log(p-value). All analyses were performed with QIAGEN IPA (QIAGEN Inc., USA)

Primary astrocyte cultures from five individual mice were exposed to either vehicle or PM_0.2_ (N = 5 biological replicates per group). A heatmap of normalized read counts is provided in Sup. Fig. S3 to visualize gene-expression patterns and hierarchical clustering across the individual PM_0.2_-exposed and vehicle-treated samples. Only DEGs with a q-value of less than or equal to 0.05 were used for the analysis. In total, we found 2217 mRNAs to be deregulated when PM_0.2_-exposed astrocytes were compared to the vehicle-treated control cells. Among these DEGs, 249 genes had log fold-change (log FC) higher than 0.5, including 76 genes with log FC greater than 1. For downregulated genes, 381 genes had log FC lower than −0.5, including 61 genes with log FC lower than −1 (Fig. 1A). A list of all the differentially expressed genes (DEGs) with log FC and q-values is included in Sup. File S2.

When we assessed the genes most upregulated after exposure, we found genes involved in xenobiotic metabolism, such as the Cytochrome P450 (CYP) genes (*Cyp1a1,* log FC = 9.23, and *Cyp1b1*, log FC = 1.74) and Aryl hydrocarbon receptor repressor (*Ahrr,* log FC = 2.96) (Sup. File S2). These genes are commonly upregulated in cells exposed to PM when the PM fraction is rich in polycyclic aromatic hydrocarbons (PAHs), which are commonly present in urban fine and ultrafine PM. We also found genes like NAD(P)H quinone dehydrogenase 1 (*Nqo1*, log FC = 3.05) and Heme Oxygenase 1 (*Hmox1*, log FC = 1.62), which are often increased in response to oxidative stress, to be upregulated after exposure. The most upregulated gene, *Cyp1a1*, log FC = 9.23, was previously reported as a blood biomarker of air pollution [44], but to our knowledge, has never been reported in PM-exposed glial cells or the brain. Of interest, we also found that the vesicular glutamate transporter 3 (VGLUT3 or *Slc17a8)* was upregulated in astrocytes with log FC = 2.10. While this transporter is primarily involved in glutamatergic transmission in neurons, in astrocytes, it is proposed to be involved in gliotransmission, the process by which astrocytes modulate neuronal activity by releasing gliotransmitters like glutamate [45]. It must be noted that evidence of VGLUT expression and function in astrocytes is sparse. Therefore, the potential effect of PM_0.2_ exposure on glutamate release by astrocytes and its consecutive effect on neuron excitability requires further investigation. Among the most downregulated genes, we found genes like Indolethylamine N-methyltransferase (*Inmt*, log FC = −3.84), that play a role in detoxification of selenium compounds and carbonic anhydrase 3 (*Car3*, log FC = −2.23), a metalloenzyme that catalyzes the reversible hydration of carbon dioxide, and many others (Sup. File S2).

### Canonical Pathways Activated by PM_0.2_ Exposure in Astrocytes

QIAGEN Ingenuity Pathway Analysis (IPA) (QIAGEN IPA, QIAGEN Inc., USA) revealed that the most activated canonical pathways were the xenobiotic metabolism pathway and nuclear factor erythroid 2–related factor 2 (NRF2)-mediated oxidative stress response (Fig. 1B**)**. By using IPA analysis, we also found that the gene *Nfe2l2* has the highest activation z-score (4.15) in our data, pinpointing a major role of the Nrf2 pathway activation. Remarkably, activation values for these pathways were quite high (z-score), as were the ratios of molecules deregulated in each pathway. For instance, the ratio value of 0.43 for xenobiotic metabolism pathway means that 43% of components of this pathway are affected by exposure, and, similarly, 26% of NRF2-mediated Oxidative Stress Response pathway genes are affected by PM_0.2_ in astrocytes (Fig. 1B). When enrichment analysis in the TRRUST database (a reference database of human transcriptional regulatory interactions) was performed [46], we found the expression of the Nrf2 transcription factor to have the highest activation values. Similarly, the activation z-score of the Nrf2 activator (4.15) in our data pinpointed the major role of Nrf2 pathway activation in the governance of astrocytes' response to PM_0.2_ exposure. Detailed pathway components deregulated after PM_0.2_ exposure in astrocytes are presented in the Sup. Fig. S4 for Nrf-2 mediated Oxidative Stress response and in Sup. Fig. S5 for Xenobiotic metabolism signalling pathway. Another most activated pathway in our data was Glutathione-mediated Detoxification, with the highest Z-score of 3.87 and half of the pathway components (53%) affected by exposure in IPA analysis (Fig. 1B). Glutathione-mediated detoxification is a key process of detoxification in astrocytes [47]. mRNA of many enzymes of Glutathione S-transferase (GST) superfamily was upregulated in our data: *Gst alpha* 13 (*Gstα13 or Gm3776*), *Gstα1*, *Gstα3, Gstα2* and *Gstα4* with log FC = 2.61, 2.59, 2.41, 2.18 and 1.36 correspondingly and *Gst µ 1 (Gstµ1)* with log FC = 1.20 (Fig. 1A, Sup. File S2). The GST superfamily is vital for cellular detoxification as it catalyzes the glutathione (GSH) conjugation to various electrophilic compounds, including xenobiotics and products of oxidative stress. The main pathway categories altered in astrocytes after PM_0.2_ exposure are presented in Fig. 1C. We found pathway categories like signal transduction, nuclear receptor signalling, and many others to be deregulated after exposure. Pathway categories connected to disease-specific pathways, cellular stress and injury, and cancer had the highest number of genes deregulated. When analysis of canonical pathways activated by exposure was performed, in addition to the activated pathways described in the previous section, we found the Aryl hydrocarbon receptor signalling pathway to be significantly inhibited. The top 20 deregulated canonical pathways are presented in Fig. 1D. The results were filtered by Z-score value, and only brain cell-related pathways were selected. The full unfiltered list of all pathways deregulated after exposure is presented in the Sup. File S3.

### Comparison of Transcriptomic Responses Between Urban PM-Exposed Astrocytes and Microglia

**Fig. 2:**
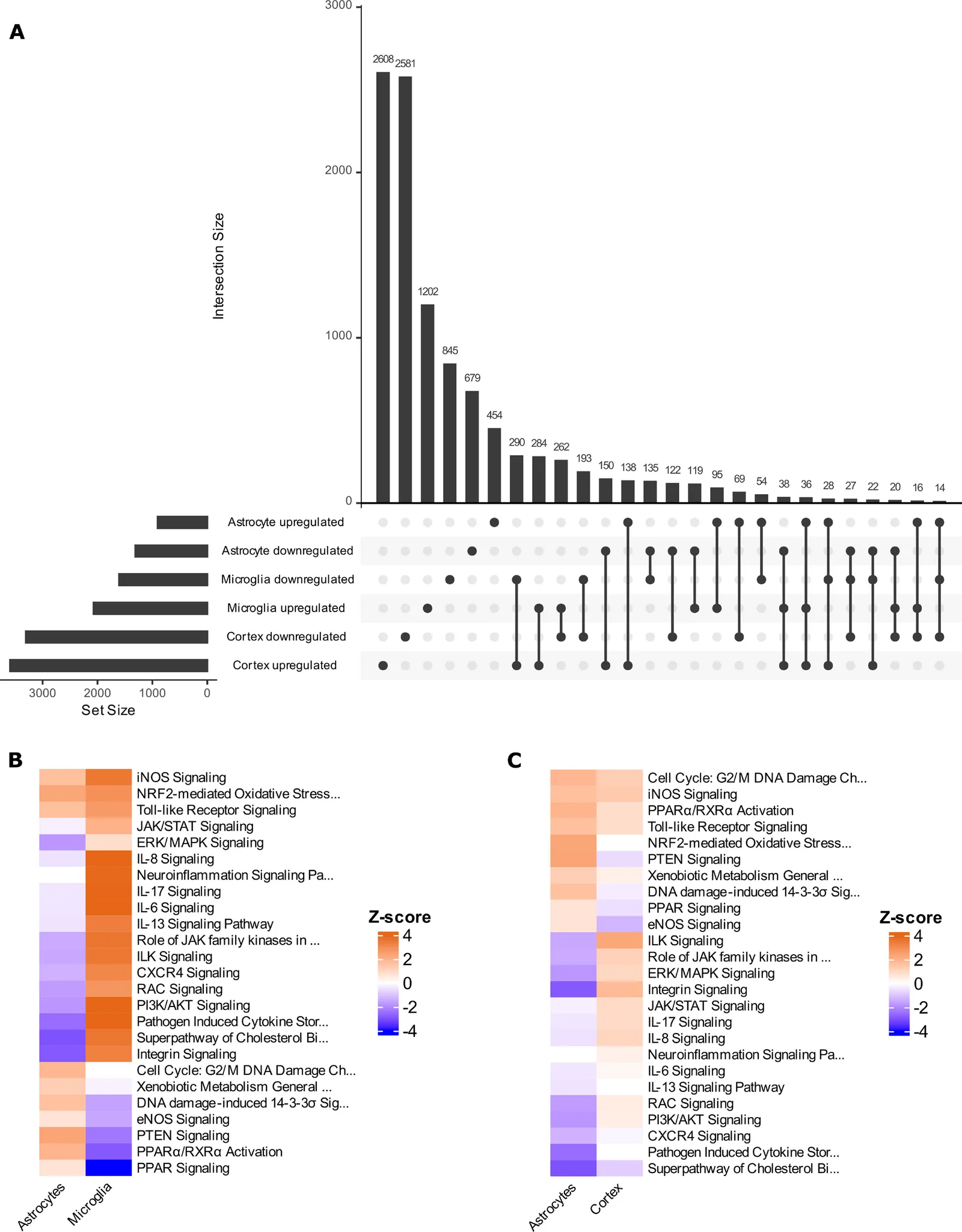
(**A**) Upset plot depicting the intersection in the lists of deregulated genes in the analysed datasets: female mouse cerebral cortex, primary mouse astrocytes and microglia exposed to PM. **B** A representative heatmap depicting selected pathways that were significantly deregulated in PM-treated microglia and astrocytes. **C** A representative heatmap depicting selected pathways that were significantly deregulated in the mouse cerebral cortex and astrocytes exposed to PM_0.2_. Only pathways with activation Z-score ≥|2| were considered for the graphs

To decipher the cell type specificity of the PM responses, we next compared our dataset with RNA-seq data from Han et al., who exposed primary mouse microglia to 100 μg/ml of urban air PM (standard reference material, SRM1648a, fine PM with diameter of 0.1–2.5 μm) [30]. In contrast, our study used 50 μg/ml of PM_0.2_. Although our study has a lower concentration, the smaller size and airborne origin of PM_0.2_ likely exert comparable or greater toxicity and, therefore, cause similar magnitude changes in gene expression. We reanalysed the raw RNA-seq data from Han et al. with the same pipeline as for our astrocyte data, enabling direct comparison of DEGs and pathway activation. We found 613 shared DEGs between the datasets. We found 135 downregulated and 95 upregulated genes shared in both PM-exposed astrocytes and microglia (Fig. 2A). IPA analysis of cell signalling pathways activated in both astrocytes and microglia after exposure was then performed. Strikingly, most of the signalling pathways were found to be highly activated in microglia in response to PM exposure, while in astrocytes, most of the cell signalling pathways were found to be downregulated. For Fig. 2B, only 25 out of 260 analysed pathways were selected. The pathways that are presented were selected based on the high activation score of greater than |2| for either or both of the cell types and relevance to the research topic. We found pathways like the Pathogen-Induced Cytokine Storm Signaling Pathway, Superpathway of Cholesterol Biosynthesis, PI3K/AKT Signaling, and many others to be highly activated in microglia but downregulated in astrocytes upon exposure. Interestingly, the Nrf2 pathway had a similar activation score in both cell types. Meanwhile, the Xenobiotic Metabolism General Pathway (Fig. 2B) and other branches of this pathway were activated only in astrocytes (Sup. File S3). On the other hand, many proinflammatory pathways like Neuroinflammation Signalling Pathway, IL-6, IL-8, IL-13, and IL-17 signalling had high activation scores in microglia, but non-significant Z-scores in astrocytes (Fig. 2B). A heat map with the top 20 deregulated pathways in astrocytes and microglia with no selection and only filtering by p-value is uploaded at Sup. Fig. S6. A full list of shared canonical pathways and their activation scores in both astrocytes and microglia is presented in Sup. File S4.

### Comparison of Transcriptomic Responses BetweenAstrocytes and the Cerebral Cortex of MiceExposed to Urban PM_0.2_

We next compared our findings with data obtained by Haghani et al., in which mice were chronically exposed to urban PM_0.2_, and then the cerebral cortex was analysed by mRNA sequencing (mRNA-seq) [31]. After we analysed both raw data from female mouse cortex obtained from the *Haghani *et al. dataset together with our data in one pipeline, we found 694 genes shared between our and *Haghani’s* DEGs, including genes like *Itgb7, Slc26a7, Il1rl1, Cxcl16,* and many others (Sup. File S5). We analysed the direction of deregulation of DEGs in both datasets, and we found 138 upregulated and 122 downregulated genes shared between PM-exposed astrocytes and cortex (Fig. 2A). The full list of common DEGs in both astrocytes and cortex is uploaded as Sup. File S5. We found pathways like iNOS signalling, PPARα/RXRα Activation, Toll-like Receptor Signaling, and others to be upregulated both in astrocytes and the cortex after exposure, depicting the common pathway response to urban PM_0.2_ (Fig. 2C). While pathways like NRF2-mediated Oxidative Stress Response, PTEN Signaling, Xenobiotic Metabolism General Signaling Pathway, DNA damage-induced 14–3-3σ Signaling, PPAR Signaling, eNOS Signaling were significantly upregulated exclusively in astrocytes and not in the cortex. A heat map with the top 20 deregulated pathways in astrocytes and cortex, with no selection and only filtered by p-value, is uploaded as Sup. Fig. S7. A full list of shared canonical pathways and their activation scores in both astrocytes and the cortex is presented in Sup. File S6.

### Changes in Protein Abundance Induced by PM_0.2_ Exposure and Integration of mRNA-Protein Data

**Fig. 3:**
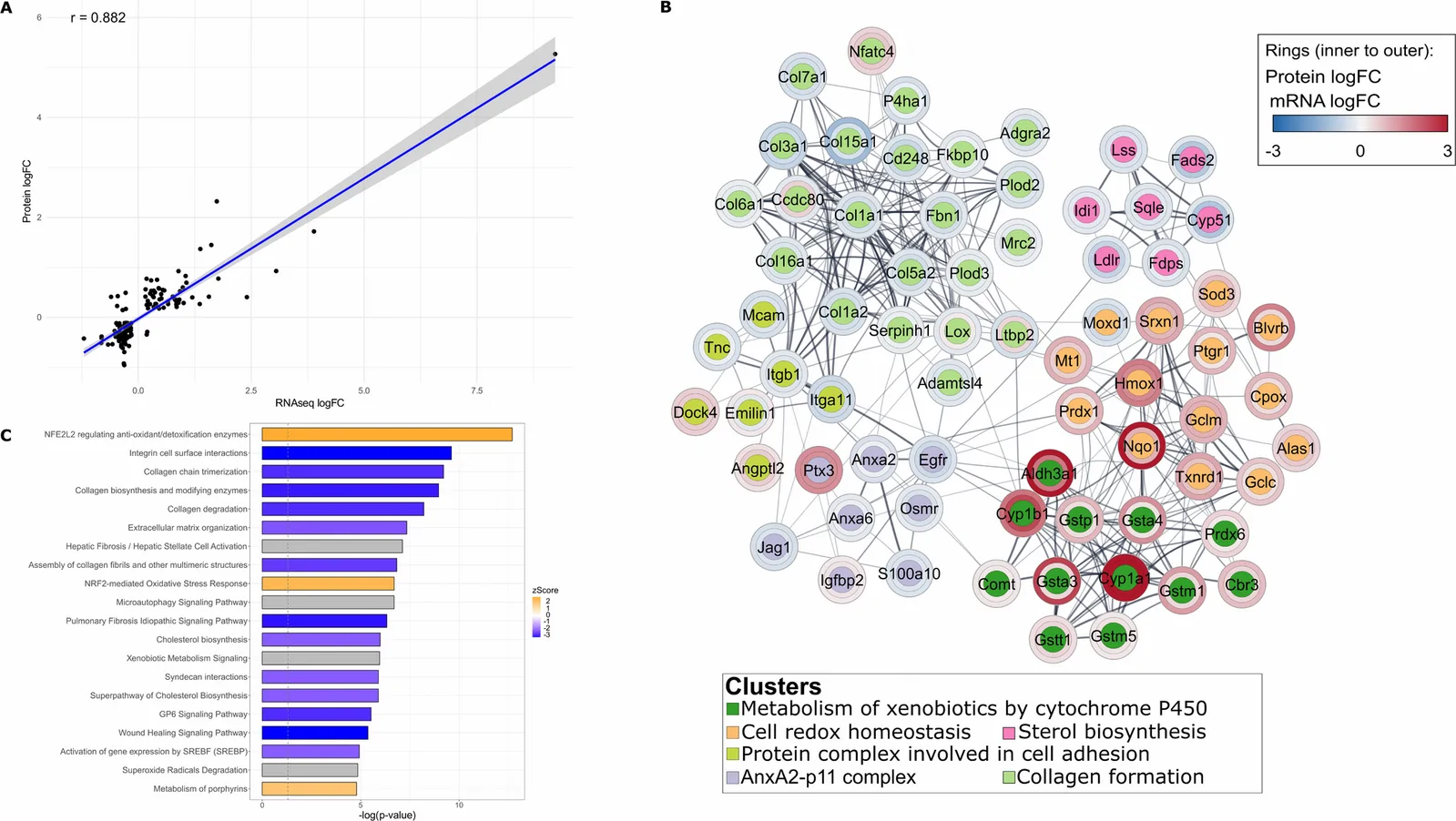
**A** Scatter plot illustrating the strong positive correlation between mRNA-seq and protein abundance log fold changes (logFC) in our experimental dataset of astrocytes. The correlation coefficient is 0.882, indicating a substantial linear relationship where increases in mRNA logFC are generally accompanied by similar increases in protein logFC.** B** Results of STRING analysis for proteins deregulated based on mRNA sequencing and proteome analysis. Protein–protein interaction network, in which inner and outer rings indicate logFC for the proteome and transcriptome, respectively. Upregulated features are shown in red, while downregulated features are shown in blue. Clusters are distinguished by the inner circle colour. **C** Exploratory IPA canonical-pathway analysis of the 142 cross-platform-supported mRNA-protein pairs. Top 20 canonical pathways deregulated in the investigated DAP subset reported by IPA software, sorted by reported p-value. Pathway colour indicates the IPA activation z-score, with positive and negative values indicating predicted activation and inhibition, respectively. The analysis was performed with QIAGEN IPA (QIAGEN Inc., USA)

With Spectronaut (v 20.4.2) identification, the total number of proteins found in astrocytes was 7566 (mean = 7423, SD = 66). After excluding contaminants and proteins with 4 or more missing values for any of the two groups, a total of 7391 proteins remained (sample mean = 7260, SD = 65), presented in Sup. File S7. Due to the relatively low N number, there were no significant DAPs after applying multiple-comparison corrections, so we opted to use the nominal p-value for DAP analysis, as previously done by [48] and discussed in [41]. Differential abundance analysis identified 354 proteins altered by PM_0.2_ exposure when using nominal p-values; the results are presented in the Sup. File S8. Hereafter, these proteins are referred to as nominally differentially abundant proteins. When comparing the proteomics data to the mRNA-seq data, among these 354 nominally differentially abundant proteins, corresponding genes of 142 of DAPs were significantly differentially expressed in the mRNA-seq dataset (Sup. File S9). The Pearson correlation coefficient between the log_2_ fold change of gene expression and protein abundance was 0.882, with a p-value < 2.2e^−16^, meaning that there is a significant high correlation between the mRNA expression and protein abundance (Fig. 3A). STRINGdb was used to visualise the protein–protein interaction network for the 142 DAPs (Sup. Fig. S8). MCL clustering performed on the network identified 25 distinct clusters; the top 6 are indicated in Fig. 3B. The most interesting clusters contained 14 (Cellular redox homeostasis) and 12 DEG-DAP nodes (Metabolism of xenobiotics by cytochrome P450), with most of the DEG-DAP nodes upregulated for each cluster, further highlighting activation of these pathways in astrocytic responses to PM_0.2_. For the Metabolism of xenobiotics by the cytochrome P450 cluster, we found proteins like CYP1A1, CYP1B1, and ALDH3A1 to be highly upregulated after exposure (log FC = 5.27, 2.32, and 1.72, respectively), consistent with their mRNA (log FC = 9.23, 1.74, and 3.89, respectively). Similarly, we observed corresponding upregulation of DEG-DAP in the Cellular redox homeostasis cluster with upregulated proteins of Hmox1, Nqo1, Gclm, Srxn1, Sod3, Txnrd1, Mt1 (with protein log FC vs. mRNA log FC = 1.45 vs 1.62, 0.93 vs 3.05, 0.93 vs 0.89, 0.83 vs 1.05, 0.75 vs 0.42, 0.70 vs 1.07, 0.52 vs 0.92, respectively) (Sup. File S9). The full result of the MCL clustering is presented in the Sup. File S10. Canonical pathway analysis was subsequently performed using the 142 cross-platform-supported mRNA-protein pairs. This exploratory analysis showed predominant inhibition, with upregulation in NRF2-related and porphyrin metabolism pathways (Fig. 3C). Interestingly, we also found pentraxin 3 (Ptx3) to be upregulated at both protein and mRNA levels after exposure, with log FC = 1.37 for both protein and mRNA.

## Discussion

Astrocytes, the most abundant glial cell type, are engaged in many pivotal functions in the brain, including the regulation of brain metabolism and BBB integrity. Among their diverse functions, astrocytes play a significant role in the brain's antioxidant defence system, protecting neural cells from the damaging effects of oxidative stress. Prior research has demonstrated that PM exposure can cause neurotoxicity and lead to gliosis, which may, in turn, affect BBB permeability and brain homeostasis [49]. However, detailed molecular insight into astrocytic responses to air pollution exposure is lacking.

### Xenobiotic Metabolism Pathway Activation

Our mRNA-seq demonstrated that the most upregulated genes after PM_0.2_ exposure were those involved in xenobiotic metabolism, such as the Cytochrome P450 (CYP) genes (*Cyp1a1,* log FC = 9.23, and *Cyp1b1*, log FC = 1.74), alongside the Aryl hydrocarbon receptor repressor (*Ahrr,* log FC = 2.96). *AHRRs* are commonly altered when cells are exposed to PAH-rich fractions, as PAHs are AHR ligands. The *Cyp1a1* gene is controlled by a bidirectional promoter with AHR response elements, making it highly inducible by various xenobiotics that are found in pollutants that act as AHR ligands. When AHR is activated, it initiates the synthesis of CYP1 enzymes that oxidize and detoxify these ligands, creating a negative feedback loop that limits further AHR signaling. In our data, we found CYP genes and Aryl hydrocarbon receptor repressor (*Ahrr,* log FC = 2.96) to be highly upregulated after exposure, and we report a significant activation of Xenobiotic Metabolism AHR Signalling Pathway, with almost half of its components of this pathway deregulated by exposure (Fig. 1B).

The CYP superfamily is an important contributor to the metabolism of both endogenous and exogenous compounds, including brain detoxification [50]. These genes respond to PAHs abundant in urban PM. For instance, *Cyp1a1* participates in the metabolism of aromatic hydrocarbons and the hydroxylation of aromatic compounds. *Cyp1b1* was previously found to be upregulated after acute exposure to DEP in a mouse cortex [7]. In our data, *Cyp1b1* was significantly upregulated in PM_0.2_-exposed mouse astrocytes at both mRNA and protein levels (log FC = 1.74 and 2.32, respectively). In our study’s sampling campaign, the PM_0.2_ fraction used for the study had the greatest mass concentrations of 5-ring and 6-ring PAHs, like phenanthrene, fluoranthene, and pyrene [28]. Our findings suggest that PAH-containing air pollutants may activate astrocytic CYP proteins, which are proposed to contribute to a protective metabolic barrier for the brain.

The shotgun proteomics analysis of PM_0.2_-exposed astrocytes strengthened the evidence provided by mRNA-seq, revealing a high correlation between DAPs and DEGs (Pearson r = 0.882). Notably, we report significant upregulation of the Metabolism of xenobiotics by the cytochrome P450 cluster, with *Cyp1a1*, *Cyp1b1* and *Aldh3a1* activation at both protein and mRNA levels, pinpointing the key involvement of the cytochrome P450 family in the detoxification response of astrocytes after air pollution exposure. Previous studies showed a greater magnitude of CYP1A1/2 induction in cultured glial cells than in neurons at both mRNA and protein levels when a well-known CYP1A-inducer was used [51]. Here, for the first time, we report a high magnitude of Cyp1a1 and Cyp1b1 induction at both mRNA and protein in response to environmental stimuli in astrocytes. Similarly, induction of aldehyde dehydrogenase family 3 member A1 (*Aldh3a1*) at both mRNA (log FC = 3.89) and protein (log FC = 1.72) level highlights activation of astrocytic aldehyde detoxification response. *Aldh3a1* oxidizes aliphatic/aromatic aldehydes to less toxic carboxylic acids, protecting against ROS-induced lipid peroxidation and was previously reported to be upregulated after cigarette smoke exposure, while *Aldh3a1* knockdown increased DNA damage and cytotoxicity [52].

### NRF2-Mediated Oxidative Stress Response

AHR-dependent induction of CYP1, AKR, NOX, and COX-2 genes can be a source of ROS generation [53]. To defend the cell against increased levels of ROS, the NRF2-mediated Oxidative Stress Response pathway is often activated. AHR signalling and NRF2 signalling interact to regulate the expression of antioxidant genes. NRF2-controlled genes such as *HMOX1*, *NQO1*, *GCLM*, and *GCLC* are often increased in response to oxidative stress induced by PM, as summarized in a recent review [54]. Our mRNA-seq data on astrocytes demonstrate significant activation of the NRF2 pathway with a Z-score of 2.56 and a quarter (26%) of its components affected by PM_0.2_ exposure when analysed in IPA software (Fig. 1B). Similarly, enrichment analysis in the TRRUST database [46] showed the highest activation values for the *Nrf2* transcription factor (4.15) in our data and pinpointed the major role of this pathway in the governance of astrocytes' response to PM_0.2_ exposure. Our findings were further confirmed with proteomics analysis, with corresponding upregulation of DEG-DAP in the Cellular redox homeostasis cluster, in which both proteins and mRNA were upregulated for *Hmox1, Nqo1, Gclm, Srxn1, Sod3, Txnrd1* and *Mt1* after the exposure. When we checked expression values for the NRF2 pathway components, we found high upregulation of *Nqo1* (mRNA log FC = 3.05, protein log FC = 0.93), *Hmox1* (mRNA log FC = 1.62, protein log FC = 1.45) and *Gclm* (mRNA log FC = 0.89, protein log FC = 0.93) that were previously reported in other cell models exposed to PM [55–57]. Furthermore, the Nrf2 pathway was found to regulate GSH production in astrocytes [58], suggesting the interconnection of the Nrf2 pathway with GSH synthesis and glutathione-mediated detoxification in the broader cellular antioxidant response system. 

### Glutathione-Mediated Detoxification

The regulation of GSH synthesis in astrocytes is critically influenced by Nrf2, which drives the expression of antioxidant defense genes, including those directly associated with GSH synthesis and release [59]. Under conditions of oxidative stress, Nrf2 activation triggers the enhanced transcription of various antioxidant genes, including the key enzymes involved in GSH synthesis, like Glutamate-cysteine ligase catalytic and modifier subunits and others [60]. Notably, astrocytes represent the primary source of GSH within the CNS [61]. GSH-mediated detoxification in astrocytes plays a central role in detoxifying a wide array of both toxic byproducts generated during normal cellular metabolic processes and environmental toxins and harmful substances in the brain, like ROS [47], various peroxides, reactive nitrogen species, carbonyls, and xenobiotics. The GSH's ability to neutralize both oxygen- and nitrogen-based radicals underscores its central role as an antioxidant in astrocytes, crucial for protecting the brain from reactive species generated during both normal cell metabolism and under stress conditions. Moreover, astrocytes contribute to neuronal antioxidant defense by supplying neurons with GSH precursors [62]. Therefore, activation of the GSH-mediated detoxification process can indicate a protective response in both astrocytes and an astrocyte-mediated protection of neurons in response to PM_0.2_. Despite the central role of the GSH-mediated detoxification pathway in brain metabolism, detoxification and response to xenobiotics, we did not find any studies reporting activation of this pathway in response to PM exposure. But we found a review that highlights the importance of GSTs in toxic metal detoxification and toxic metal-mediated neurodegeneration [63].

In our data, the glutathione-mediated detoxification pathway had the highest activation Z-score of 3.87, with half of the pathway components affected (Fig. 1B). In detail, multiple GST superfamily enzymes were upregulated, including *Gstα1–4, Gsta13* and *Gstµ1* (log FC range: 1.20–2.61; Sup. File S1). GSTs catalyze the GSH conjugation to various electrophilic compounds, including xenobiotics, carcinogens, and products of oxidative stress, enabling their detoxification and efflux.

### Comparison of Astrocytes and Microglia Responses to Urban PM

Since data on the comparison of astrocyte PM-induced responses to other brain cell types is sparse, we compared our dataset with mRNA-seq on mouse primary microglia exposed to 100 μg/ml of urban fine PM from SRM1648a (0.1–2.5 μm diameter) [30]. Our study used 50 µg/ml PM_0.2_ (≤ 0.2 µm), which, despite the twofold lower concentration, likely elicits comparable transcriptional effects due to its higher toxicity from smaller size and real-world airborne composition. Reanalysis using a unified pipeline identified 613 shared DEGs between PM-exposed astrocytes and microglia.

Canonical pathway analysis revealed striking cell type differences. The super pathway of Cholesterol Biosynthesis had the highest activation score in microglia (z-score = 3.46) but was inhibited in astrocytes with almost the same magnitude (Z-score = −3.0). Microglia play an important role in brain cholesterol de novo synthesis, metabolism and trafficking [64], while astrocytes play a pivotal role in the remyelination process through Nrf2-mediated astrocyte-oligodendrocyte interaction [65]. Inhibition of the Nrf2 pathway was reported to increase cholesterol biosynthesis in astrocytes [65]. Here, we show that PM_0.2_ exposure results in both significant activation of the Nrf2 pathway and robust inhibition of the superpathway of cholesterol biosynthesis in astrocytes. Altogether, these data align with PM-linked white matter pathology and corpus callosum atrophy reported previously [66–68], indicating myelin damage upon PM exposure.

Many of the proinflammatory pathways, such as the Pathogen-Induced Cytokine Storm Signaling Pathway (z-score = 5.17 in microglia and −1.99 in astrocytes), Neuroinflammation Signaling Pathway (4.65 vs. 0.16, respectively), or IL-17 signalling (4.75 vs. −0.41, respectively), were upregulated in mouse microglia but had a low Z-score in astrocytes. IL-17 activation drives downstream signaling, including pro-inflammatory cytokines like IL-1β, IL-6, and IL-8 [69], which were robustly activated in PM-exposed microglia (IL-6 z-score = 4.08; IL-8 = 5.34) but suppressed in astrocytes (−0.60 and −0.51).

The Nrf2 pathway had a similar activation score in both cell types, showing the universal cytoprotective role of this pathway, as reported in many studies showing Nrf2 activation in response to environmental stimuli. Interestingly, we found that the lysosomal CLEAR (Coordinated Lysosomal Expression and Regulation) pathway showed opposing regulation (z-score = 1.85 in astrocytes and −1.9 in microglia). Meanwhile, the xenobiotic metabolism general pathway (Fig. 3B) and other branches of this pathway were activated only in astrocytes (Sup. File S4). These divergences highlight astrocytes' bias toward detoxification versus microglia's proinflammatory profile in PM responses.

### Comparison with the Cerebral Cortex of PM_0.2_ -Exposed Mice

A comparison of our mRNA-seq data with cortical samples from chronically urban PM_0.2_-exposed mice [31] revealed 694 shared DEGs, including, e.g. *Itgb7, Slc26a7, Il1rl1, Cxcl16,* and many others (Sup. File S5). These overlaps indicate common transcriptional responses to PM_0.2_ across brain cell types and exposure durations.

To note, several pathways were significantly upregulated exclusively in astrocytes and not in the cortex, including NRF2-mediated Oxidative Stress Response, PTEN Signaling, Xenobiotic Metabolism General Signaling Pathway, PPAR Signaling, and eNOS Signaling, potentially reflecting cell type-specific contributions to detoxification.

All in all, our findings promote astrocytes as important contributors to the brain's defence against xenobiotics. Robust activation of NRF2-oxidative stress response genes, xenobiotic metabolism and GSH-mediated detoxification pathways components in our study suggests that astrocytes may contribute to brain detoxification responses. We suggest that while microglia may be the primary instigators of neuroinflammation in response to PM, astrocytes primarily contribute to detoxification. We suggest activation of a tripartite mechanism, synergistically activating the NRF2-mediated oxidative stress response, xenobiotic metabolism pathways, and GSH detoxification pathways, ultimately working to metabolise ROS and xenobiotics in both astrocytes themselves and to protect vulnerable neurons from PM-induced toxicity and oxidative stress.

### Study Limitations

Several study limitations temper broader interpretations and must be reported. The study used cultures from adult female mice only, limiting data on sex differences or male astrocyte responses to PM. A single dose concentration and 24 h exposure time-point limit extrapolation to longitudinal exposures and do not provide data on dose-specific transcriptomic and proteomic responses. The direct comparison between real-life urban PM exposure and the airborne PM applied in vitro is not possible: acute in vitro exposure does not model the repeated, chronic, and multicellular exposure conditions experienced in real-world scenarios. The present study aimed to identify astrocyte-intrinsic mechanisms activated by an acute PM exposure, rather than to reproduce chronic human ambient exposure. Therefore, our findings should be interpreted as a mechanistic discovery of astrocyte-intrinsic molecular responses to airborne nPM rather than modelling of real-world exposure conditions. Future studies using repeated low-dose exposures, aerosol-based deposition systems, and in vivo models are required to better define the environmental relevance and long-term consequences of these responses. Because no DAPs met the Benjamini-Hochberg-adjusted significance threshold, the nominally differentially abundant proteins and related analyses should be interpreted as exploratory and require confirmation in larger independent cohorts. Comparative analysis with prior studies is limited by available datasets and confounded by methodological disparities (no proteomics data, different PM type used in the studies). While the data support the hypothesis of astrocytic detoxification responses in the exposed brain, the hypothesis requires further in vivo validation. In addition, the reported omics data should be further validated with functional assays and tested in different models, such as a co-culture exposure model. Although the independent transcriptomic and proteomic datasets consistently identified PM-associated alterations, the present study did not establish the functional consequences or causal roles of the identified pathways. Future studies should use genetic and pharmacological perturbation of selected NRF2, xenobiotic-metabolism, and glutathione-detoxification pathway components, combined with functional assays to determine whether the observed molecular responses represent adaptive protection, cellular stress, or mechanisms that contribute to PM-induced cytotoxicity.

## Conclusions

This paper demonstrates astrocyte-specific gene- and protein-level responses to urban nanoscale PM_0.2_ exposure, with 2217 DEGs and 354 DAPs, confirming a coordinated response at both transcriptomic and proteomic levels. Pathways related to xenobiotic metabolism, Nrf2-mediated oxidative stress response and GSH-mediated detoxification were strongly induced by PM_0.2_ exposure in astrocytes. Proteomics integration revealed high mRNA-protein concordance (r = 0.882) and STRING clusters enriched for redox homeostasis and CYP-mediated xenobiotic metabolism, highlighting astrocytes’ role in brain xenobiotic defense and protection against PM-derived PAHs.​​ Cell type comparisons pinpointed this specialization: unlike PM-exposed microglia, which preferentially activate proinflammatory pathways, astrocytes suppress inflammation while activating AHR signalling and GSH-mediated detoxification pathways. Shared Nrf2 pathway activation in both glial cells underscores its universal cytoprotection role.​ The discovered overlaps with chronically PM_0.2_-exposed mouse cortex (694 shared DEGs) suggest some conserved brain-specific responses to PM, yet astrocyte-enriched pathways like PPAR Signaling, eNOS Signaling, and others suggest cell-specific contributions that are overlooked in bulk tissue analysis. Collectively, these findings identify an astrocyte-specific transcriptomic response to 24 h PM_0.2_ exposure in primary cultures derived from adult female mice. Future studies at the single-cell level, and studies integrating functional perturbations and in vivo models are warranted to confirm these findings and to establish whether the identified pathways have a protective role or contribute to PM-induced neurotoxicity.

## Supplementary Information

Below is the link to the electronic supplementary material.

ESM 1 (XLSX 1.84 MB)

ESM 2 (DOCX 2.41 MB)

## Data Availability

The RNA-sequencing data have been submitted to the European Nucleotide Archive (ENA) under the accession number PRJEB90554. The mass spectrometry proteomics data have been deposited to the ProteomeXchange Consortium via the PRIDE partner repository with the dataset identifier PXD065183.
