# Supplementary material for: Urban PM0.2 Affects Astrocytes by Inducing Xenobiotic and Oxidative Stress Responses
Source: Mol Neurobiol. 2026 Oct 2;63(1):942. doi: 10.1007/s12035-026-06232-w (PMC13633141; doi:10.1007/s12035-026-06232-w)
Supplement: Supplementary file 1 [file 12035_2026_6232_MOESM2_ESM.docx]

**Supporting information for:**

**Urban PM_0.2_ Affects Astrocytes by Inducing Xenobiotic and Oxidative Stress Responses**

Liudmila Saveleva^1^, Alexey Afonin^1^, Mariia Ivanova^1^, Michal Sima^2^, Zdenek Krejcik^2^, Tarja Malm^1^, Pasi I Jalava^3^, Pavel Rössner^2^, Katja M Kanninen^#1^

*^1^A.I. Virtanen Institute for Molecular Sciences, University of Eastern Finland, Kuopio, 70211, Finland*

*^2^Department of Toxicology and Molecular Epidemiology, Institute of Experimental Medicine CAS, Prague, Czech Republic*

*^3^Department of Environmental and Biological Sciences, University of Eastern Finland, Kuopio, 70211, Finland*

*^#^ Corresponding author, +358 46 9210065;* [*katja.kanninen@uef.fi*](mailto:katja.kanninen@uef.fi)*, University of Eastern Finland, A.I. Virtanen Institute for Molecular Sciences, Kuopio Campus, Bioteknia, Neulaniementie 2, P.O. Box 1627, FI-70211 Kuopio, Finland*

**Sup. Fig. S1.** Dose-dependent effects of PM_0.2_ and vehicle exposure on viability of primary mouse astrocytes. Cultures from four independent mice were exposed to vehicle or PM_0.2_ (25–500 µg/mL) for 24h, followed by MTT assay. Data presented as mean ± SD (n = 4 biological replicates per group; each replicate is an average of 6 technical replicates). One-way ANOVA was used for comparison among multiple groups, followed by Dunn's post hoc test: ***p* < 0.001 vs. untreated control.

**Sup. Fig. S2.** Principal component analysis (PCA) plot showing transcriptomic similarity between the cells used in this study and reference astrocytes and microglia transcriptomes from the dataset of Pan et al. (<https://pubmed.ncbi.nlm.nih.gov/32238175/>). Both untreated control samples (green) and nPM-treated astrocytes (dark orange) from this study cluster distinctly along the first principal component (PC1), which explains 70% of the total variance. Their expression profiles align more closely with the astrocyte transcriptome (yellow) than with microglial data (blue) from Pan et al in the PC1 axis. Separation from both reference datasets is primarily driven by the second principal component (PC2), which explains 28% of the total variance.

**Sup. Fig. S3.** Heatmap with normalized read counts showing sample clustering. Samples M1C-M5C are control vehicle; M1T-M5T-PM_0.2_**-**exposed astrocytes.

**Sup. Fig. S4**. Nrf2 pathway activated by exposure in PM_0.2_**_-_**exposed astrocytes. Red - genes that are upregulated in our data; orange - genes predicted to be activated in this pathway; green – downregulated genes in our data.

**Sup. Fig. S5**. Xenobiotic pathway activated by exposure. Red - genes that are upregulated in our data, orange – genes that are predicted to be activated in this pathway, green – downregulated genes in our data, blue – genes that are predicted to be inhibited in the pathway.

**Sup. Fig. S6**. A representative heatmap depicting the top 20 significantly deregulated pathways (by p-value) in PM-treated microglia and astrocytes.

**Sup. Fig. S7**. A representative heatmap depicting the top 20 pathways (by p-value) that were significantly deregulated in astrocytes treated with PM_0.2_ for 24h and in the cerebral cortex of mice chronically exposed to PM_0.2_ (Haghani et al., 2020).

**Sup. Fig. S8**. Results of STRING analysis for proteins deregulated based on mRNA sequencing and proteome analysis. Protein-protein interaction network, in which inner and outer rings indicate logFC for the proteome and transcriptome, respectively. Upregulated features are shown in red, while downregulated features are shown in blue. Clusters are distinguished by the inner circle colour, with grey circles representing clusters 6 to 13.
